# Host Taxon Predictor - A Tool for Predicting Taxon of the Host of a Newly Discovered Virus

**DOI:** 10.1038/s41598-019-39847-2

**Published:** 2019-03-05

**Authors:** Wojciech Gałan, Maciej Bąk, Małgorzata Jakubowska

**Affiliations:** 1 Faculty of Biochemistry, Biophysics and Biotechnology, Jagiellonian University in Kraków, ul. Gronostajowa 7, 30-387 Kraków, Poland; 2 AGH University of Science and Technology, Faculty of Materials Science and Ceramics, al. Mickiewicza 30, 30-059 Kraków, Poland

## Abstract

Recent advances in metagenomics provided a valuable alternative to culture-based approaches for better sampling viral diversity. However, some of newly identified viruses lack sequence similarity to any of previously sequenced ones, and cannot be easily assigned to their hosts. Here we present a bioinformatic approach to this problem. We developed classifiers capable of distinguishing eukaryotic viruses from the phages achieving almost 95% prediction accuracy. The classifiers are wrapped in Host Taxon Predictor (HTP) software written in Python which is freely available at https://github.com/wojciech-galan/viruses_classifier. HTP’s performance was later demonstrated on a collection of newly identified viral genomes and genome fragments. In summary, HTP is a culture- and alignment-free approach for distinction between phages and eukaryotic viruses. We have also shown that it is possible to further extend our method to go up the evolutionary tree and predict whether a virus can infect narrower taxa.

## Introduction

The rise of next generation sequencing (NGS) has made metagenomics a gold standard in microbial ecology. Despite its limitations, mainly lack of universal marker genes between viruses, viral metagenomics became the main tool for virus discovery^1^. Metagenomic approaches enabled virus discovery without the need of cultivation. On the other hand, the lack of well-specified host-virus cocultures hinder identification of the host of a newly discovered virus. To bypass this limitation several experimental methods have been elaborated. For example, lytic phages might be discovered and assigned to their host by the use of fosmids from natural communities^2^. Select phages could be associated with their hosts employing phageFISH, but this method requires prior knowledge of host gene marker sequences^3^. Another approach, viral tagging, links wild viruses with their host using staining with a fluorescent dye and flow-cytometric sorting but is limited to cultured hosts^4^.

The process of virus-host linkage is not only carried out with wet laboratory techniques but bioinformatic tools become more widely recognized and utilized for that purpose. Select tools focus on searching for prophages in bacterial genomes, for example Phaster^5^, PhiSpy^6^, Phage_Finder^7^, Prophinder^8^. Although in this instance the phage-host relationship is obvious, this approach is limited to phages whose genomes are, in some life cycle phases, incorporated into the host and whose host has been already sequenced. Therefore, many other computational approaches to predict the phage’s host exist. The computational methods to predict the host on phylum, class, order, family, genus, and species level have been reviewed and benchmarked extensively by Edwards *et al*.^9^ The following subjects were covered in the assessment: host-phage abundance in metagenomes, sequence homologies resulting from acquisition by phages of some host genome fragments, signatures of previous viral infections in CRISPR system, and host’s and phage’s oligonucleotide profiles comparison. Later, Zhang *et al*.^10^ reported successful application of some machine learning approaches to predict phage’s host on genus level using oligonucleotide frequencies as sequence features - including: logistic regression, support vector machines, random forest, Gaussian naive Bayes, and Bernoulli naive Bayes. Comparing phage’s and host’s oligonucleotide profiles was leveraged by application of more sophisticated similarity measures by Ahlgren *et al*.^11^ Besides investigation of phage-host relationship, tools for detection of viral signals in metagenomic data are available and progressively extended e.g. VirSorter^12^, VirFinder^13^, and MARVEL^14^.

The aforementioned approaches were applied to investigate host-phage relationship (in our study we use the term ‘phages’ to refer to viruses that infect Bacteria and Archaea), but the problem of inferring host of eukaryotic viruses is less studied. In 2010 Kapoor *et al*. reported the discovery of three novel Picorna-like viruses, and announced a new method to infer the taxa of their hosts. The technique, which they named the nucleotide composition analysis (NCA), utilizes differences in the nucleotide composition between the viruses infecting distinct hosts. Viruses were represented by their mono- and dinucleotide frequencies as well as dinucleotide bias. The bias was assessed as a ratio between the observed frequency of a dinucleotide and its expected frequency determined by multiplying the frequencies of the two constituent mononucleotides. Using the discriminant analysis, the group was able to distinguish between the viruses infecting mammals, plants, and insects with 96% prediction accuracy^15^. Although the application of the method was limited to one, albeit huge, group of viruses, it gained interest in the scientific community, and similar methodology was later applied to infer host of some (mainly ssRNA) eukaryotic viruses^16–21^. Unfortunately, the authors have not released any tool that could reinforce attempts to predict the taxon of the host of a newly sequenced virus. Some groups have investigated different machine learning, alignment, and alignment-free *k*-mer frequency dissimilarity – based approaches in predicting viral host, but their studies were limited only to some groups of viruses^11,22–25^.

In our study we first aimed to answer a more general question and develop classifiers capable of distinguishing between phages and non-phages. Viruses constituting the currently known taxa, to the authors’ best knowledge, infect either eukaryotes or Bacteria/Archaea but never both groups. Therefore, this task may seem straightforward with sole use of BLAST alignment^26^. However highly divergent viruses may lack sequence similarity to any previously sequenced virus^27^, and new viral genera are still discovered (for example Pandoravirus^28^). Moreover, various biological material, for example fecal samples, contain phages as well as eukaryotic viruses^15^, thus the origin of a sample does not help to determine whether a newly sequenced virus is a phage or not. Here we present a novel tool that helps in this respect. Host Taxon Predictor (HTP) is a versatile tool in sense of target group of viruses and is able to predict whether a virus infects eukaryotes or Bacteria/Archaea with very high accuracy. The source code of HTP is available on https://github.com/wojciech-galan/viruses_classifier.

In the course of the study, viral nucleotide sequences were represented by simple sequence features (mono-, dinucleotide absolute frequencies and di-, trinucleotide relative frequencies, see *Methods* section) and nucleic acid type. Four supervised machine learning methods were used to predict taxon of viral hosts. These methods, when trained with [object, class] pairs, allow for assigning a class to a previously unseen object. Logistic regression (LR) looks for the best fitting model to describe the relationship between the class and features of an object, and transforms the model’s output using the sigmoid function to return class probabilities^29^, k-Nearest Neighbors (kNN) is an algorithm that classifies an object to a class by a majority vote of its k nearest neighbors^30^. Quadratic Discriminant Analysis (QDA) creates decision boundaries between classes based on a combination of object’s features. Then it assigns the object to a class depending on which side of the boundary the object falls^31^. Support Vector Classification (SVC) creates a hyperplane between object belonging to two classes in a way that maximizes the margin between the classes. Moreover, it is able to map input features into higher dimensional feature space^32^. SVC is considered one of the best classification methods but is computationally expensive, while LR, QDA, and kNN are computationally cheaper but less accurate. We also tested multiple subsets of viral sequence features in a search for ‘core’ features that are responsible for infecting a specific group of organisms. Since the very high accuracy of phage vs non-phage prediction we aimed to extend our study and create models that are able to distinguish viruses infecting seed plants, vertebrates, and arthropods and obtained well-predictive models in the first two cases. Further, we used several feature selection approaches to determine which sequence features are the most important for classification.

We have also checked HTP’s behaviour on a collection of newly discovered viruses. HTP performs well either on full-length sequence or subsequences that resemble the long contigs obtained in metagenomic studies. Unfortunately, HTP shows weak performance on shorter subsequences analogous to short contigs or sequence reads.

## Results

### Classifiers designed to discriminate between phages and eukaryotic viruses

Complete viral reference genomic sequences were downloaded from NCBI Nucleotide on October 11, 2017. For the classification purposes, we employed four machine learning techniques (SVC, kNN, LR, and QDA) which are able to assess the probability that given object belongs to a distinct class, in this case, *Eukaryota*-infecting or phage. When binarized, the probabilities are transformed to exact class. Performance of such classification techniques could be measured in many ways. Accuracy, a percentage of correct predictions relative to all predictions made is the simplest measure of classification performance. However, when the cardinality of classes differ significantly, classification accuracy may be misleading. Let us consider a collection containing 90 objects of type A, 10 object of type B and a dummy classifier predicting always A regardless of the object. Despite being completely impractical, the classifier would show 90% classification accuracy when assigning objects’ types. In the case of two-class classification, some methods devoid of this disadvantage exist. Two of them were employed in this study: Matthew’s Correlation Coefficient^33^ (MCC) that measures classification performance taking into account exact classes; thus, applicable after threshold-dependent binarization of the class probabilities, and area under the receiver operating characteristic curve^34^ (AUC) which is threshold-independent. Relying on these two parameters, we designed a ‘fitness’ measure that maximizes the classifier’s performance for a typical binarization threshold = 0.5 as well as classification in a threshold-independent manner, and which does not to vary too much depending on the training data (Equation (3), *Methods* section). AUC values were interpreted as in Roelen *et al*.^35^ (AUC = 0.9–1.0 represents excellent, AUC = 0.8–0.9 good, AUC = 0.7–0.8 fair, and AUC = 0.6–0.7 poor classifier).

The classification task was performed based on the virus nucleotide sequence features (nucleic acid type and mono-, di-, and trinucleotide frequencies) only. All viruses were represented as 101 elemental feature vectors composed of sequence features. Performance of classifiers trained with various hyperparameter values and feature sets was compared using cross-validation. The cross-validation allows us to select classifiers’ hyperparameters and feature subsets exhibiting optimal usability in our classification tasks.

We hypothesized that some of the initial features may introduce noise and lead to declined performance of the classifiers. To verify the hypothesis, we applied several techniques to select the features that allow for the best classifiers’ performances. In the case of logistic regression, we used Lasso regularization which forces regression coefficients corresponding to certain features to be set to zero^36^. Lasso regularization is not applicable to the three remaining machine learning methods. In the case of kNN and QDA, we used genetic algorithms as well as our own bottom-up approach. Since SVC is much more computationally expensive than the kNN and the QDA, we applied a different feature selection techniques in this case: selecting k best features (SelectKBest) according to the highest features’ scores^37^, recursive feature elimination (RFE)^38^ Smoothly Clipped Absolute Deviation^39^ (SCAD), and quick permutation test (QuiPT)^40^. The feature-selection approach possesses also additional benefits - it helps to determine ‘core’ features responsible for the ability to infect a specific host and survive inside the host’s body.

**Figure 1:**
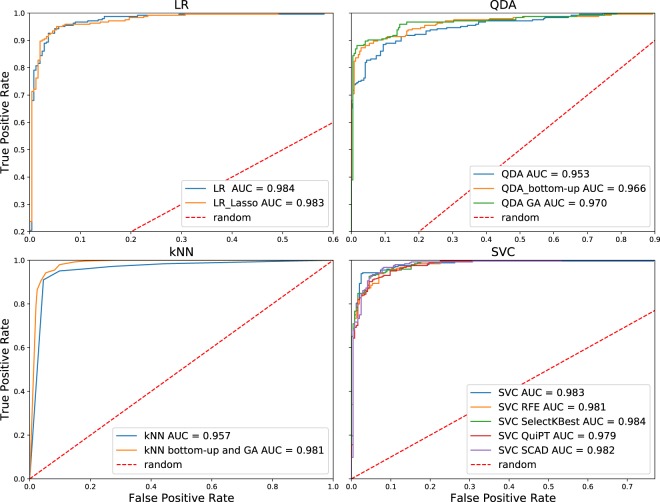
Receiver operating characteristics curves calculated on the test dataset for LR, QDA, kNN and SVC algorithms, respectively. The phages were treated as a positive class. True Positive Rate (TPR) and False Positive Rate (FPR) values essential to compute the AUCs are available in a serialized form at https://github.com/wojciech-galan/supplementary_materials/blob/master/datasets/ROC_test_set_phages_vs_rest.dump.

Feature sets and algorithm hyperparameters determined in the course of the feature selection step might have been fitted to the cross-validation data; therefore, their actual performance needed to be evaluated on the separate test set. This set comprised feature vectors that do not belong to the cross-validation data. As shown in the Table 1 and Fig. 1, all of the mentioned classifiers exhibit excellent prediction capabilities, with AUC exceeding 0.95 and prediction accuracy reaching up to 94.5%, as measured on the test set. Performance of LR classifier remains almost identical after feature selection, kNN’s and QDA’s increased, and SVC’s slightly decreased in terms of AUC. These observations supports our hypothesis that, in the case of kNN and QDA, some of the initial features impair the performance of the classifiers. Interestingly, in case of kNN, the two feature selection approaches that we applied, resulted in the same set of features. On the other hand, genetic algorithms selected features that improved classification with QDA in comparison to our bottom-up approach. All of the feature selection approaches that we applied to SVC turned up to pick features that show similar classification efficacy in terms of AUC, MCC, and accuracy.

**Table 1:** Comparison of the performance of the classifiers designed to distinguish between phages and *Eukaryota*-infecting viruses.

Classifier and the method of feature selection	Classifier’s performance estimated using cross-validation			Classifier’s performance measured on the test set			
	Average accuracy	Average MCC	Average AUC	Accuracy	MCC	AUC	Number of features
LR, no feature selection	0.897	0.805	0.946	0.945	0.889	0.984	101
LR, Lasso feature selection	0.909	0.826	0.960	0.945	0.89	0.983	49
kNN, k = 5, no feature selection	0.83	0.697	0.927	0.893	0.794	0.957	101
kNN, k = 9, bottom-up, genetic algorithms	0.935	0.878	0.980	0.936	0.874	0.981	17
QDA, no feature selection	0.920	0.853	0.970	0.887	0.778	0.954	101
QDA, bottom-up	0.972	0.945	0.986	0.918	0.838	0.966	37
QDA, genetic algorithms	0.978	0.957	0.986	0.928	0.858	0.970	28
SVC, no feature selection	0.895	0.801	0.939	0.943	0.885	0.983	101
SVC, RFE	0.921	0.847	0.965	0.926	0.852	0.981	32
SVC, SelectKBest	0.908	0.825	0.953	0.939	0.877	0.984	95
SVC, QuiPT	0.916	0.842	0.956	0.926	0.853	0.979	58
SVC, SCAD	0.918	0.843	0.959	0.934	0.869	0.982	60

When selecting features for kNN, both of the applied approaches returned the same feature set.

### Sequence features that allow the distinction between phages and non-phages

The selected feature subsets are summarized in Supplementary Table ST1. Since two of the feature selection approaches resulted in identical feature subsets, the feature subsets were later treated as one. Each of the possible sequence feature appeared in at least one feature subset. Two of the features: molecule type (DNA/RNA) and relative GGA frequency were present in all eight feature subsets, while relative frequencies of TG, TAT, TGT, TTA, and TTC in all but one subset. Features’ abundances among the selected subsets are summarized in Supplementary Table ST2. The probability, that the number of common features obtained by intersecting random feature subsets of the same lengths that the ones obtained by feature selection would be equal or greater than two, is ≈3 *10^−3^, which indicates non-random nature of the selected feature subsets (see also Supplementary Fig. SF1). Feature weights after feature selection are shown in Supplementary Table ST3. Molecule type, when DNA is encoded as positive and RNA as negative number, exhibits strong positive contribution to phages over all of the feature weights. Contrarily, relative CGA frequency shows positive contribution to eukaryotic viruses. Interestingly, feature weights obtained using various approaches show at least moderate positive correlation (Supplementary Fig. SF2).

### Classifiers designed to identify plant-, vertebrate-, and arthropod-specific viruses

**Figure 2:**
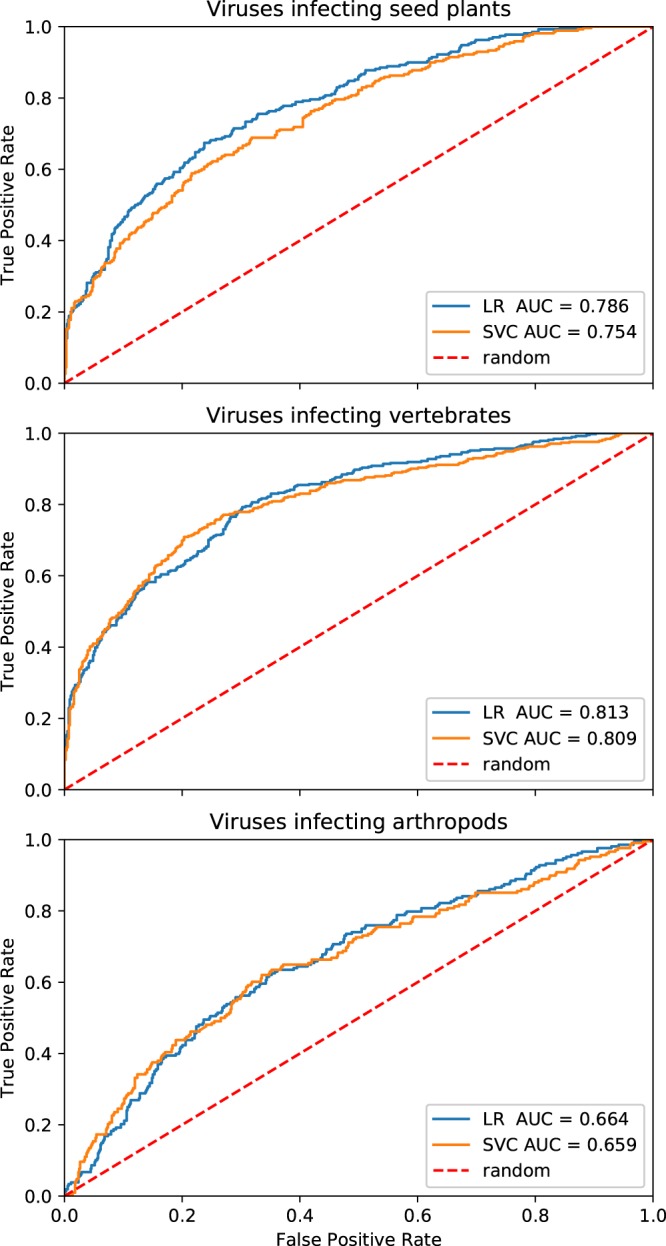
Receiver operating characteristics curves calculated on the test dataset consisting of eukaryotic viruses when detecting viruses infecting seed plants, vertebrates and arthropods, respectively. TPR and FPR values essential to compute the AUCs are available in a serialized form at https://github.com/wojciech-galan/supplementary_materials/blob/master/datasets/eukaryotic_viruses_fpr_tpr.dump.

Considering very good results of applying machine learning approach to distinguish between phages and non-phages, we investigated whether the predictive characteristics of our models could be extended to the more distant branches of the evolutionary tree. Since the problem of phage-host relationship has been studied extensively (see *Introduction*) we aimed to look for the possibility of distinction between various groups of eukaryotic viruses. For that purpose we decided to use a simplified approach: only SVC and LR due to their optimal results when applied to distinguish between phages and non-phages, only two feature selection methods: RFE and Lasso, and average AUC instead of ‘fitness’ measure to achieve classifier performance in cross-validation. We performed three separate binary classification tasks to identify viruses infecting seed plants, vertebrates, and arthropods. Our approach allowed to obtain predictors that exhibited good classification properties (AUC > 0.8) for viruses infecting seed plants and vertebrates (Fig. 2, Table 2). Unfortunately, we obtained only poor (AUC < 0.7) classification efficacy when we tried to identify arthropod-specific viruses.

**Table 2:** Comparison of the performance of the classifiers designed to distinguish between different groups of eukaryotic viruses.

Group of viruses	Classifier	Average AUC estimated using cross-validation	AUC measured on the test set
Viruses infecting seed plants	LR, Lasso	0.744	0.824
	SVC, RFE	0.738	0.754
Viruses infecting vertebrates	LR, Lasso	0.772	0.814
	SVC, RFE	0.824	0.809
Viruses infecting arthropods	LR, Lasso	0.707	0.664
	SVC, RFE	0.737	0.659

### Host Taxon Predictor software performance

**Figure 3:**
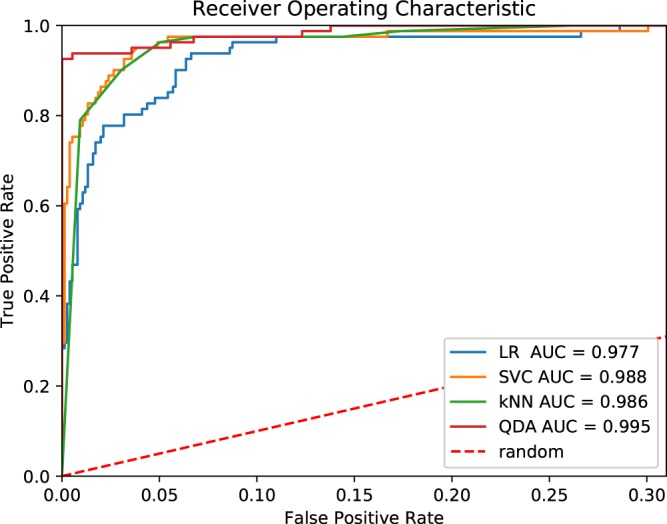
Receiver operating characteristics curves calculated for HTP’s performance evaluation on newly published viral genomic sequences. TPR and FPR values essential to compute the AUCs are available in a serialized form at https://github.com/wojciech-galan/supplementary_materials/blob/master/datasets/new_viruses_fpr_tpr.dump.

We utilized four of our classifiers designed to distinguish between phages and non-phages (LR, kNN, QDA, and SVC trained with features selected with Lasso, bottom-up, genetic algorithms, and RFE approaches, respectively) to create a Python console application that we named Host Taxon Predictor (HTP). HTP’s performance was then evaluated on complete viral genomic sequences that were published in NCBI Nucleotide after October 11, 2017 (thus after we had downloaded the initial collection of sequences that the classifiers were trained on). Each of the sequences was analyzed to obtain class probabilities. Since the classes were highly imbalanced (number of phages was about 10x lower than eukaryotic viruses, see *Methods*), we used two different evaluation metrics: AUC and MCC. As demonstrated on Fig. 3 and Table 3, all of available classifiers show very good predictive capabilities, but much better in regard to AUC (>0.97) than MCC (>0.68), even considering different ranges of the two metrics.

**Table 3:** HTP’s classification performance on newly published viral genomic sequences.

Classifier	AUC	MCC
LR	0.977	0.701
SVM	0.988	0.812
kNN	0.986	0.685
QDA	0.995	0.913

AUC and MCC were computed separately for all of the classifiers available in HTP.

**Figure 4:**
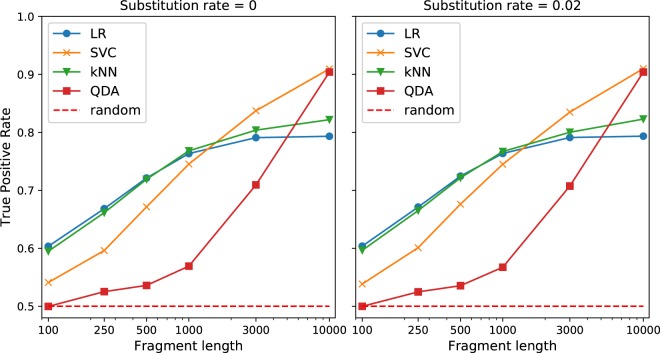
AUC as a function of subsequence length for no substitution and substitution rate = 0.02. Substitution corresponds to mutations and/or sequencing errors. The substitution rate, that we have examined, almost does not change HTP’s performance. TPR and FPR values essential to compute the AUCs are available in json format at https://github.com/wojciech-galan/supplementary_materials/blob/master/datasets/results_for_stimulated_metagenomics.json.

Encouraged by successful application of HTP’s on complete genomic sequences, we evaluated its performance on shorter sequence fragments also. We utilized the same collection of newly published viral sequences to generate subsequences of lengths 100, 250, 500, 1000, 3000, and 10000, which corresponded to the range of sequence read/contig lengths obtained by next generation sequencing and subsequent genome assembly techniques. Select viruses exhibit high mutation rate^41^ and numerous NGS platforms tend to generate reads with relatively high error rate^42^, both mutation and errors are represented in our study by substitutions. The probability of generating a subsequence from a sequence was proportional to the sequence length (see Methods). Since phages’ genomic sequences tend to be longer than most of the eukaryotic viruses’ ones, number of generated sequences belonging to both classes was similar.(Supplementary Table ST4). HTP exhibits good predictive capabilities for long subsequences but fails for shorter ones (Fig. 4, Supplementary Table ST5 and Supplementary Fig. SF3). We did not observe any considerable effect of the analyzed substitution rate on HTP’s performance (respective AUC values varied by <0.005). LR and kNN tend to show fair (AUC > 0.7) predictive capabilities for at least 500 nucleotide long subsequences. SVC exhibits the fair predictive performance for at least 1000 nt but for longer sequences outperforms both kNN and LR. QDA shows AUC > 0.7 only for 10000 nt long subsequences but for this fragment length outperforms both LR and kNN and also shows best prediction for complete sequences (Table 3). Good prediction (AUC > 0.8) is possible only for subsequence lengths ≥ 3000.

## Discussion

To the authors’ best knowledge, our study is the first that attempts to distinguish between phages’ and eukaryotic viruses’ sequences in an alignment-independent manner. Our solution does not require prior knowledge of a host’s sequence. In our study, we aimed to create an application neither limited to any group of viruses nor any group of hosts. First, we created classifiers capable of such distinction. The classifiers use only simple sequence features (nucleic acid type, mono-, di-, trinucleotide abundances). Feature subsets and classifier hyperparameters, resulting in the best distinction, were selected using cross-validation set and final classifiers were later evaluated on a separate test set. The test set provided an unbiased evaluation of a final model fit on the training dataset. Obtained results indicate excellent prediction capabilities of our classifiers. Along with feature extraction code, the classifiers were wrapped in one Python console application that we named Host Taxon Predictor. We chose those classifiers’ hyperparameters and feature sets that performed best on cross-validation data.

We later evaluated HTP’s performance on a collection of complete viral genomes that were published in the NCBI Nucleotide database after we had created our cross-validation and test sets. The additional inspection confirmed that HTP is able to properly classify newly sequenced viruses. We also wanted to investigate, whether HTP can make correct predictions on short sequence fragments of different lengths that resemble contigs or reads obtained during metagenomic studies. We received good classification results for at least 3000 nt-long, fair for at least 500 nt-long, and poor for shorter subsequences.

Being encouraged by the satisfactory results of distinction between phages and eukaryotic viruses, we tested a similar approach on higher branches of the evolutionary tree. Since the problem of phage-host relationship is studied extensively, we aimed to distinguish between several groups of eukaryotic viruses. We succeeded in recognizing’ plant and vertebrate viruses, but fell short of identifying viruses that infect arthropodes. In summary, we created a proof of concept which functionalities can be potentially extended by machine learning experts through plugging new classifiers. To the authors’ best knowledge, our study is the first that manage to distinguish between eukaryotic viruses infecting various host taxa and is not limited to only a group or several groups of viruses. Other analysis considered only, for example, picorna-like viruses^15^, influenza virus^25^, or several groups of RNA viruses^22,24^. An important part of such extension should be not only training a novel classifier but also selection of the most appropriate feature sets. As we shown (Table 1), feature selection boosted classifiers’ performances on cross-validation data. When examined on test set, classifier’s performance slightly decreased for SVC but increased in half of the tested machine learning algorithms - kNN and QDA. These results indicate slight overfitting when models were trained with all the available features. It is important to note that the feature selection techniques were necessary in our study. The number of feature combinations $({\sum }_{k=1}^{101}\frac{101!}{k!\ast (101-k)!}\approx \,2.54\ast {10}^{30})$ that rise from 101 features greatly exceeds our computational resources. Nevertheless, since two feature selection approaches resulted in exactly the same set of features we believe it is reasonable to assume that the utilized methods converged to a useful solution for this classification task. We also demonstrated moderate to high linear correlation between feature weights for various classification algorithms and feature selection approaches. This indicates that generally feature weights are similar, and features discarded by one approach are frequently discarded by another one. Further, the possibility of obtaining two features when intersecting random feature sets is very low, which is an evidence of our feature sets being selected in a non-random way.

Better prediction properties for longer oligonucleotides’ abundances have been already shown^10^ and our preliminary results support this thesis (Supplementary Notes). However, we decided to limit our feature set to mono-, di-, and trinucleotides, because the number of features substantially increases for longer nucleotides. Inclusion of tetranucleotides to our feature set would increase the number of possible feature combinations to ${\sum }_{k=1}^{357}\frac{357!}{k!\ast (357-k)!}\approx \,2.94\ast {10}^{107}$. As a result, some of our feature selection approaches could converge to lower local maxima or would become impractical due to computational complexity. Inclusion of hexanucleotides would result in a greater number of features than a number of elements in our dataset, thus increase in the risk of overfitting.

Nucleic acid type is the most important sequence feature for distinction between phages and eukaryotic viruses as it appeared in all of the feature subsets. Having genetic information stored in the form of DNA strongly increases the probability of a virus to be classified as a phage. It is not surprising, because most of the known phages are actually DNA viruses (only 18 of 975 and 5 of 244 phages in our cross-validation and test set, respectively, are RNA viruses). The underrepresentation of RNA phages is most probably an effect of undersampling of viral diversity, rather than a general tendency among *Viridae*. For example, most of metagenomic studies focused on DNA only and could not evaluate novel RNA bacteriophages^43^. We also checked predictive capabilities of nucleic acid type alone (Supplementary Figs SF4 and SF5), but except for the shortest subsequences (≤250 nt) the results were much worse compared to the best of our classifiers.

Another feature that appeared in all of the feature subsets was GGA relative frequency. It shows positive contribution on a virus to be classified as eukaryotic. We did not find clear explanation of this observation in the literature. It is important to note that mononucleotides tend to be less abundant than di- and trinucleotides (present in, on average, 2.25 subsets versus 3.94 and 3.64 subsets, respectively) and relative dinucleotide frequency more abundant than absolute frequency in the selected feature subsets (4.19 versus 3.89 subsets). TA as well as CG underrepresentation was previously reported to be characteristic for various groups of eukaryotic viruses (for example^44–46^) but frequencies of these dinucleotides did not match the most abundant (present in 7 or 8 feature sets) sequence features. TA absolute frequency was present in 6 of 8 selected feature subsets, and shows negative contribution on classification as a phage. Possible origins of TA underrepresentation across genomes have been summarized by Di Giallonardo *et al*.^45^, but the importance of this dinucleotide in predicting viruses infecting this particular group of hosts is yet to be discovered. TAT and TTA relative frequencies, in which TA is included, are present in 7 feature subsets, but the relative trinucleotide frequency was designed to be independent from constituent dinucleotides (see Equation (2), Methods). CG relative frequency is present in 5 and absolute frequency only in 3 feature sets. These observations suggest that, when classifying viruses using multiple sequence features, individual CG and TA frequencies might not be so important, as it was previously implied, and are consistent with more recent studies^46,47^. We did not discover any coded amino acid pattern between the most abundant features.

Although measuring computational performance of various feature selection approaches was out of scope of our study, we observed that QuiPT was much faster than other methods. Albeit binarization of the features required by QuiPT leads to loss of information, we obtained features similarly useful for classification as with other methods. Likewise, our bottom-up feature selection approach resulted in the same (kNN) or similarly performing (QDA) feature sets in addition to being quicker.

Similarly to the authors of VirSorter and VirFinder, we investigated the performance of our software on viral genomes published after construction of our original dataset. Since the analysis included about 10 times more eukaryotic viruses than phages, MCC is an appropriate metric for classifier evaluation. In such case AUC metric may be too optimistic^48^. MCC and AUC are not directly comparable, because MCC returns values between −1 and 1, while AUC between 0 and 1. When MCC values obtained during the classification are transformed to [0, 1] range, they give more conservative evaluation results than AUC but even in the worst case (kNN, 0.8425) indicate a good classifier. Moreover, the transformed MCC values for SVC and QDA indicate excellent classifier (0.906 and 0.956, respectively) and confirms HTP’s applicability for phage versus non-phage classification.

When analyzing sequence fragments, both classes were similar in number, so the strong class imbalance was no longer an issue, and we used only AUC which is one of the most widely used measures for binary classifiers^49^. Similarly, we used only AUC metric when distinguishing between eukaryotic viruses infecting distinct groups of hosts.

Popular short-read NGS platforms produce 35–700 base long reads, while the long-read ones - many kilobases long^42^. The reads are later assembled into longer contigs. Unfortunately, HTP cannot be applied to short reads/contigs, below 500 bases. For longer, 500–1000 base long reads/contigs, we suggest applying HTP with LR and for 1000–3000 base long ones kNN classifier, but results obtained for <3000 bases should be taken with caution. 3000 base long reads/contigs allow reliable classification with SVM and QDA classifier but the latter shows slightly worse results. Taking into consideration also the results obtained for complete sequences, we suggest using QDA classifier for complete or almost complete genomes. When sequencing error rate is expected to be higher than 2%, HTP’s result also should be taken with caution. HTP’s weak performance on short sequences may be a result of our training procedure. VirFinder’s authors demonstrated relationship between length of the training sequences and classification performance when distinguishing between phages’ and hosts’ sequences of different lengths^13^. Classification performance was the highest when the query sequence length matched the sequence length on which the model was trained. Our classification task is different, but future training our models on shorter sequences may also improve HTP’s classification of short ones. We also consider application of neural networks as an interesting direction of research. Application of deep learning architectures in biology receives increasingly growing interest and dramatically improved the state-of-the-art in many fields. Prediction of a viral host may particularly benefit from use of recurrent neural networks, designed for utilizing sequential data^50^.

Our application does not detect and remove non-viral sequences. When a researcher expects presence of such sequences, they should be filtered out prior to the application of HTP. Some tools capable of performing such analysis (VirSorter, VirFinder, MARVEL) exist, but their application is limited to distinction between bacteria and phages. VirFinder may potentially misclassify eukaryotic sequences as viral^13^. Nevertheless, as VirFinder’s authors stated, their tool could potentially be trained to distinguish also eukaryotic viruses.

Compositional similarities between viruses’ and their host’s genomes have been reported among phages and, to some extent, eukaryotic viruses^51–53^. The similarities were also confirmed by Roux *et al*. when analyzing viral sequences mined from bacterial and archaeal genomic data^54^. Ahlgren *et al*. examined possibility of application of different oligonucleotide- based distance/dissimilarity measures to predict the host of a bacteriophage and obtained accuracies of 33% at the genus to 75% at the phylum level^11^. Contrary to the studies, Di Giallonardo *et al*. demonstrated that dinucleotide composition has better predictive power when inferring family of an animal virus than its host^45^. VirFinder’s authors reported successful application of oligonucleotide frequency and logistic regression to distinguish between phages and their hosts. Taken together with machine learning approaches to predict a host of a virus, that we mentioned in the Introduction section, these results lead to conclusion that viral genomic sequences can contain various signals specific for a host and/or a viral family.

Some viruses are not easily culturable, so they could not be discovered in the pre-metagenomic era^55^, and many of the sequences discovered using metagenomic approaches could not be identified as viral, because of being distant and unalignable to the already known viruses^27^. Thus, databases of viral sequences may be biased and so our cross-validation and test sets as well as additional collection of newly sequenced viruses. Despite being alignment-independent, machine learning approaches to classify viral sequences assume that viruses infecting distinct host taxa share certain (oligo-)nucleotide usage pattern concealed at first glance. As new taxons of viruses are being discovered it may turn out that the patterns utilized by our classifiers are not shared among large groups of newly identified viruses and, therefore, the classifiers would need update.

## Summary

Constant reduction of sequencing costs has accelerated the pace of viral discovery. Today, new viruses are identified in environmental samples (water, air, sewage), as well as feces and body fluids. These samples could potentially contain viruses infecting a broad range of hosts. For example, human feces contain viruses infecting humans as well as recently eaten plants and animals, and bacteria inhabiting human gut^56^. In the case of an outbreak of a previously unknown disease, it might be very important to distinguish the viruses that could actually infect an organism of interest from contaminants. This applies not only to humans but plants and farm animals as well. At their current state, our classifiers are able to distinguish the phages, thus consequently can facilitate elimination of vast majority of targets when searching for gut pathogens (the most of human gut viruses are phages^57^). They could also be applied to search for therapeutic phages in human excretions.

## Methods

### Data collection and determination of host’s lineage

Viral nucleotide reference sequences were retrieved from the NCBI Nucleotide database on October 11, 2017. Besides the sequence itself, the files in XML format often contain information about viral lineage, natural hosts, nucleic acid type (DNA/RNA), number of strands, etc. In the process of metadata verification, the naming convention of hosts was verified and supplemented with Latin names in case of a common name usage. In certain cases, if a user submitted a common name of the host that appeared ambiguous (e.g. ‘sugarcane’) or was not present in the NCBI Taxonomy (‘mulberry’), the proper Latin name was supplied manually. We have developed a script that utilizes the host’s name to extract its full lineage from the NCBI Taxonomy database. The host lineage subsequently served to assign a virus to a class (‘phage’, ‘eukaryote-infecting’, ‘vertebrates-infecting’, etc.).

### Feature extraction

Each sequence was transformed into a vector of viral sequence features: mono- and dinucleotide frequencies, relative di- and trinucleotide frequencies (all of which calculated for a single strand) and nucleic acid type. To compute relative di- and trinucleotide frequencies we applied the formulas developed by Burge *et al*.^58^ excluding components related to reverse strands. Thus, relative frequency of XY dinucleotide (ρ_XY_) is a ratio between its absolute frequency (f_XY_) and frequencies of its components, f_X_ and f_Y_:

1 $${\rho }_{XY}=\frac{{f}_{XY}}{{f}_{X}\ast {f}_{Y}}$$

while relative trinucleotide frequency γ_XYZ_ is built to account for all first- and second order effects:

2 $${\gamma }_{XYZ}=\frac{{f}_{XYZ}}{{\rho }_{XY}\ast {\rho }_{YZ}\ast {\rho }_{XNZ}\ast {f}_{X}\ast {f}_{Y}\ast {f}_{Z}}=\frac{{f}_{XYZ}\ast {f}_{X}\ast {f}_{Y}\ast {f}_{Z}}{{f}_{XY}\ast {f}_{YZ}\ast {f}_{XNZ}}$$

where XNZ is a trinucleotide, and N in f_XNZ_ and ρ_XNZ_ represents any nucleotide. The features were extracted using our original script and standardized in order for each feature to have zero-mean and unit-variance.

As a result of the previous procedures, we obtained 5275 feature vectors that could be easily split by lineage into two subsets: viruses infecting eukaryotes and those infecting prokaryotes and *Archaea*, comprising 4056 and 1219 viruses, respectively. Each vector consisted of 101 elements (4 mononucleotide frequencies, 16 dinucleotide frequencies, 16 relative dinucleotide frequencies, 64 trinucleotide frequencies and nucleic acid type).

### Data partitioning

For further analysis, a balanced set composed of all non-*Eukaryota*-infecting viruses and an equal number of viruses that infect eukaryotes were prepared. 80% of data (975 viruses from each groups) was later used for cross-validation to select best models’ hyperparameters, while the remaining 244 viruses from each group were utilized as an independent test set. The goal was to partition the viruses between cross-validation and test set so that both sets would contain dsDNA, dsRNA, ssRNA+, ssRNA−, dsRNA viruses as well as retroviruses while preventing redundancy between the sets (similar viruses should not appear in both sets). The partitioning was performed automatically in regard to viral lineage. The process is described in detail in Supplementary Notes.

Partitioning viruses between cross-validation groups was performed separately for phages and viruses infecting eukaryotes. Phages were sorted alphabetically along their lineages and then divided into 5 subsets. The sorting along viruses’ lineages prevents redundancy between the subsets of the cross-validation set. The initial dataset contained larger number of *Eukaryota*-infecting viruses. Thus, in order to balance virus categories, a different strategy to select a virus to one of the cross-validation splits was employed. The viruses were arranged according to their lineages, and a random sample of viruses of a given lineage were selected to the cross-validation set. The probability of picking an *i-th* virus of the given lineage to the cross-validation set *P(v*_*i*_) is determined by the equation $P({v}_{i})={e}^{-{i}^{0.217}}$. The number 0.217 was earlier determined using the *minimize* function from scipy.optimize^59^ to ensure that the number of *Eukaryota*-infecting viruses assigned to the cross-validation set is close to the number of phages assigned to this set. Eventually, 975 viruses were picked and divided into 5 subsets just as in the case of phages.

### Machine learning – general approach

The cross-validation set, comprising 1950 (80%) viruses, was applied to select features and/or model hyperparameters, while the test set (488 viruses) used for model evaluation. The 5-fold stratified cross-validation was applied to obtain mean AUC and MCC along with a standard deviation of these parameters. The best classifier was then determined as the one with maximal value of ‘fitness’ measure

3 $$fitness=\overline{MCC}+\overline{AUC}-{\sigma }_{MCC}/4-{\sigma }_{AUC}/4$$

where $\overline{MCC}$ and $\overline{AUC}\,$stand for mean MCC and AUC, while σ_MCC_ and σ_AUC_ for their standard deviations. In this study, MCC was always computed at 0.5 threshold.

Four machine learning algorithm implementations were employed for classification purposes: logistic regression, SVC with linear kernel, kNN, and QDA from Python scikit-learn (sklearn) package. For the classification without feature selection, default sklearn parameters except for linear kernel in SVC were used (for details, please refer to Supplementary Notes).

### Feature selection: logistic regression

Numerous strategies were tried for the selection of the set of features that maximizes classifier’s performance. Eventually, the Lasso regularization was used to select best features when applying logistic regression. The regularization parameter C ranged from 2^−5 to 2^5 as consecutive powers of two.

### Feature selection: kNN and QDA

In the bottom-up approach, all possible feature combinations of length one to three were evaluated. The resulting initial feature sets were sorted according to their ‘fitness’ value. In the next step, one of the highest-scoring 100 feature sets was quasi-randomly determined as the basis for appending subsequent features. Our preliminary studies demonstrated that randomness was essential for the algorithm not to converge too early to a local ‘fitness’ maximum. Assuming *l* is the length of the feature set, in the next step all possible feature sets of length *l* + 1 were generated, and their ‘fitnesses’ were evaluated. For each of new feature sets (length *l* + 1), if any of its subsets of length *l* had not already been evaluated, it was also appraised during this step. Then, again, all sets were sorted according to their ‘fitness’ value, and the process was repeated, starting with the second step until no new feature set appears in the 100 highest-scoring ones during consecutive 1000 rounds. The algorithm along with more detailed description is illustrated in the Supplementary Fig. SF6. In the case of kNN feature selection with bottom-up approach was performed independently for k = 1, 3, 5, 7, and 9.

In second approach, genetic algorithms (GA) with tournament selection implemented in the Python DEAP package^60^ were used. Each feature set was represented as an ordered vector of 101 binary attributes, while each attribute corresponds to the presence/absence of a given feature in the feature set. The grid search strategy was adopted to run the feature selection on a small population of 100 individuals (binary vectors) to choose best GA settings. Following an analysis of initial run results, the grid of GA settings was extended in the most promising directions. Finally, the best settings to run GA on bigger population of 500 individuals was chosen. In each case, evolution was terminated after 200 generations or when all individuals were identical. Each analysis was repeated five times, and the individual that maximizes the ‘fitness’ measure was selected for further analysis. GA setup is described in detail in Supplementary Notes.

### Feature selection: SVC

In the case of SVC, different feature selection techniques were applied: selecting k best features (SelectKBest from scikit-learn) - according to the highest values for the ANOVA F-value between a label and a feature with k ranging from 1 to 101, RFE from scikit-learn^61^, SCAD from penalizedSVM R-package^62^, and QuiPT from biogram R-package^40^. All of analyses were performed using SVC with linear kernel and equal range of C parameter values, as in the case of logistic regression with Lasso regularization. In process of selecting features with biogram, only those with p-value < 0.01 were considered.

### Evaluation of the final models

Following the selection of the best model hyperparameters and/or the best feature sets using cross-validation, the final model was trained with all data from the cross-validation set and evaluated on the test set. To be implementation-independent, the evaluation was performed exclusively using sklearn^61^. For the tested models, we computed MCC, classification accuracy and AUC, along with a false positive rate (FPR) and a true positive rate (TPR) for given thresholds.

### Comparing our feature selection approaches with selecting random features

A simple set intersection was made to determine common features among selected feature vectors. To confirm that common features did not originate from random selection, feature subsets comprising of arbitrary features were generated and an intersection of subsets made. Numbers of features in these subsets were equal to the cardinalities of the original feature subsets. This action was repeated 10^6^ times and, as a result, a distribution of numbers of common elements in the intersections was obtained. The way of creating the distribution imply full independence between contents of particular subsets.

Pearson correlation coefficients between features weights along with 2-tailed p-values were computed with scipy^59^.

### Distinction between viruses infecting seed plants, vertebrates, and arthropods

To check whether it is possible to distinguish between several groups of eukaryotic viruses utilizing the same sequence features as in the previous analyses, a following approach was used. The set of 4056 eukaryotic viruses was divided into four groups: those infecting seed plants, vertebrates, arthropods, and other viruses (infecting fungi, simple deuterostomes, etc.), consisting of 1147, 1175, 1143, and 591 viruses, respectively. Subsequently, viruses were partitioned between cross-validation and test sets similarly as in previous analyses. No balancing of classes was implemented, i.e. every eukaryotic virus was certainly assigned to either cross-validation or test set. Finally, the test set consisted of about 23% viruses (270, 371, and 208 viruses infecting seed plants, vertebrates, arthropods, respectively and 78 other eukaryotic viruses, in total 927). The partitioning is described in detail in Supplementary Notes.

The host prediction was transformed to three binary classification tasks, and we checked separately for virus’ target group (seed plants, arthropods, vertebrates) or lack of it. Feature selection with Lasso regularization (logistic regression) and recursive feature elimination (linear SVC) was performed similarly as for distinction between eukaryotic viruses and phages with the use of AUC in place of ‘fitness’ measure.

### Viral Feature Extractor and Host Taxon Predictor software

Scripts for downloading viral genomic sequences, extracting viral and host lineage, and transforming sequences into features, written in Python 2.7, were merged together in one package named Viral Feature Extractor.

For all of machine learning algorithms (LR, QDA, kNN, SVC), hyperparameters and feature sets exhibiting the highest ‘fitness’ on cross-validation data were adopted to train the final classifiers using all the feature vectors from cross-validation as well as test set. The classifiers were wrapped with feature extraction functions in one Python console application called Host Taxon Predictor. The application accepts algorithm name, nucleic acid type, and raw or fasta-formatted viral genomic sequence and returns either the exact class of virus (*Eukaryota*-infecting or phage) or class probabilities.

### Host taxon predictor evaluation on new viruses

A new collection of viral nucleotide reference sequences was downloaded on August 27, 2018 and hosts’ lineages were determined as described in Data collection and determination of host’s lineage sections. Host’s lineages for 837 new viruses were determined, of which 756, 72, and 9 infects *Eukaryota*, *Bacteria*, and *Archaea*, respectively. The viral sequences were later analyzed by HTP to obtain class probabilities and compute AUC and MCC. Identifiers of the viral sequences are available at https://github.com/wojciech-galan/supplementary_materials/blob/master/datasets/new_viruses_ids.

### Host taxon predictor evaluation on sequence fragments

The newly downloaded sequence collection was used to evaluate HTP’s performance on sequence fragments. The generation of sequence fragments datasets was attempted with WGSIM simulator (https://github.com/lh3/wgsim) which crashed with fragment length > 1000 nt. The challenge was addressed by creation of authors’ own script which is available at https://github.com/wojciech-galan/supplementary_materials/blob/master/code/my_read_simulator.py. The script was applied to produce sequence fragments of lengths *s* and base substitution rate *r*, where *s* equals to 100, 250, 500, 1000, 3000 or 10000, and *r* equals to 0 or 0.02. The sequence fragments were generated randomly. Probability of generating a subsequence from a sequence of length *S* was proportional to *S*-*s* + 1 for *S*-*s* ≥ 0, so a subsequence could be generated only if the subsequence is supposed to be no longer than the original sequence. Next, sequence fragments were analyzed with HTP as described above.

## Supplementary information

HostTaxonPredictor_supplementary_info

## Data Availability

Viral Feature Extractor and Host Taxon Predictor are available as git repositories at https://github.com/wojciech-galan/Viral_feature_extractor and https://github.com/wojciech-galan/viruses_classifier, respectively. To support the idea of transparency in science and allow everyone to reproduce our results we have released also our datasets as well as scripts that we used to obtain particular results. They are available on https://github.com/wojciech-galan/supplementary_materials. Stable versions of these repositories are accessible on zenodo under 10.5281/zenodo.2531529, 10.5281/zenodo.2531545, and 10.5281/zenodo.2531541, respectively.
