# Supplementary material for: Host Taxon Predictor - A Tool for Predicting Taxon of the Host of a Newly Discovered Virus
Source: Sci Rep. 2019 Mar 5;9:3436. doi: 10.1038/s41598-019-39847-2 (PMC6400966; doi:10.1038/s41598-019-39847-2)
Supplement: Supplementary file 1 — HostTaxonPredictor_supplementary_info [file 41598_2019_39847_MOESM1_ESM.docx]

Title: **Host Taxon Predictor - a tool for predicting taxon of the host of a newly discovered virus.**

Authors: Wojciech Gałan, Maciej Bąk, Małgorzata Jakubowska

*Supplementary Table ST1: Features selected with various feature selection approaches*

| Feature selection approach | Selected features |
| --- | --- |
| LR, Lasso | molecule, AA frequency, CA frequency, CC frequency, AG relative frequency, CC relative frequency, GA relative frequency, GC relative frequency, GG relative frequency, TA relative frequency, TG relative frequency, TT relative frequency, AAG relative frequency, ACC relative frequency, ACG relative frequency, ACT relative frequency, AGA relative frequency, AGC relative frequency, AGG relative frequency, ATC relative frequency, CAA relative frequency, CAC relative frequency, CAG relative frequency, CCC relative frequency, CCT relative frequency, CGC relative frequency, CGG relative frequency, CGT relative frequency, CTA relative frequency, CTG relative frequency, GAA relative frequency, GAC relative frequency, GAT relative frequency, GCC relative frequency, GCT relative frequency, GGA relative frequency, GGC relative frequency, GTA relative frequency, GTC relative frequency, TAC relative frequency, TAG relative frequency, TAT relative frequency, TCA relative frequency, TGG relative frequency, TGT relative frequency, TTA relative frequency, TTC relative frequency, TTG relative frequency, TTT relative frequency |
| SVC, SCAD | molecule, A frequency, AA frequency, AC frequency, AG frequency, AT frequency, C frequency, CA frequency, CC frequency, CG frequency, CT frequency, G frequency, GA frequency, GC frequency, GG frequency, GT frequency, TC frequency, AA relative frequency, AT relative frequency, CC relative frequency, CT relative frequency, GC relative frequency, GT relative frequency, TA relative frequency, TC relative frequency, TG relative frequency, AAG relative frequency, AAT relative frequency, ACA relative frequency, ACG relative frequency, AGA relative frequency, AGC relative frequency, ATA relative frequency, CAC relative frequency, CAT relative frequency, CCA relative frequency, CGT relative frequency, CTA relative frequency, GAA relative frequency, GAC relative frequency, GAG relative frequency, GCA relative frequency, GCC relative frequency, GGA relative frequency, GGC relative frequency, GGG relative frequency, GTA relative frequency, GTC relative frequency, GTG relative frequency, TAC relative frequency, TAT relative frequency, TCA relative frequency, TCT relative frequency, TGA relative frequency, TGC relative frequency, TGG relative frequency, TGT relative frequency, TTA relative frequency, TTC relative frequency, TTT relative frequency |
| SVC, QuiPT | molecule, AC frequency, AG frequency, CA frequency, CC frequency, CG frequency, CT frequency, GA frequency, GC frequency, TA frequency, TG frequency, AA relative frequency, AG relative frequency, CA relative frequency, CC relative frequency, CG relative frequency, CT relative frequency, GA relative frequency, GC relative frequency, GG relative frequency, TG relative frequency, AAA relative frequency, AAG relative frequency, AAT relative frequency, AGA relative frequency, AGG relative frequency, ATG relative frequency, CAC relative frequency, CCG relative frequency, CCT relative frequency, CGA relative frequency, CTA relative frequency, CTC relative frequency, CTG relative frequency, CTT relative frequency, GAA relative frequency, GAC relative frequency, GAG relative frequency, GCC relative frequency, GCT relative frequency, GGA relative frequency, GGC relative frequency, GGG relative frequency, GGT relative frequency, GTA relative frequency, GTC relative frequency, TAC relative frequency, TAG relative frequency, TAT relative frequency, TCC relative frequency, TCT relative frequency, TGA relative frequency, TGC relative frequency, TGG relative frequency, TGT relative frequency, TTA relative frequency, TTC relative frequency, TTT relative frequency |
| SVC, RFE | molecule, CA frequency, CC frequency, GG frequency, TA frequency, AG relative frequency, CC relative frequency, GA relative frequency, GC relative frequency, TA relative frequency, TG relative frequency, TT relative frequency, ACG relative frequency, AGA relative frequency, CAG relative frequency, CGT relative frequency, CTA relative frequency, GAA relative frequency, GAG relative frequency, GAT relative frequency, GCC relative frequency, GGA relative frequency, GGC relative frequency, GGG relative frequency, GTC relative frequency, TAG relative frequency, TAT relative frequency, TGG relative frequency, TGT relative frequency, TTA relative frequency, TTG relative frequency, TTT relative frequency |
| SVC, SelectKBest | molecule, A frequency, AA frequency, AC frequency, AG frequency, AT frequency, CA frequency, CC frequency, CG frequency, CT frequency, G frequency, GA frequency, GC frequency, GG frequency, GT frequency, T frequency, TA frequency, TC frequency, TG frequency, AA relative frequency, AC relative frequency, AG relative frequency, AT relative frequency, CA relative frequency, CC relative frequency, CG relative frequency, CT relative frequency, GA relative frequency, GC relative frequency, GG relative frequency, GT relative frequency, TA relative frequency, TC relative frequency, TG relative frequency, TT relative frequency, AAA relative frequency, AAC relative frequency, AAG relative frequency, AAT relative frequency, ACA relative frequency, ACC relative frequency, ACG relative frequency, AGA relative frequency, AGC relative frequency, AGG relative frequency, AGT relative frequency, ATA relative frequency, ATC relative frequency, ATG relative frequency, ATT relative frequency, CAA relative frequency, CAC relative frequency, CAT relative frequency, CCC relative frequency, CCG relative frequency, CCT relative frequency, CGA relative frequency, CGG relative frequency, CGT relative frequency, CTA relative frequency, CTC relative frequency, CTG relative frequency, CTT relative frequency, GAA relative frequency, GAC relative frequency, GAG relative frequency, GAT relative frequency, GCA relative frequency, GCC relative frequency, GCG relative frequency, GCT relative frequency, GGA relative frequency, GGC relative frequency, GGG relative frequency, GGT relative frequency, GTA relative frequency, GTC relative frequency, GTG relative frequency, GTT relative frequency, TAA relative frequency, TAC relative frequency, TAG relative frequency, TAT relative frequency, TCA relative frequency, TCC relative frequency, TCG relative frequency, TCT relative frequency, TGA relative frequency, TGC relative frequency, TGG relative frequency, TGT relative frequency, TTA relative frequency, TTC relative frequency, TTG relative frequency, TTT relative frequency |
| QDA, bottom-up | molecule, G frequency, GT frequency, T frequency, TA frequency, TG frequency, AA relative frequency, AC relative frequency, AG relative frequency, CG relative frequency, TC relative frequency, AAT relative frequency, AGA relative frequency, AGC relative frequency, ATC relative frequency, ATG relative frequency, ATT relative frequency, CAT relative frequency, CCA relative frequency, CTC relative frequency, CTT relative frequency, GAG relative frequency, GGA relative frequency, GGG relative frequency, GGT relative frequency, GTG relative frequency, GTT relative frequency, TAA relative frequency, TAT relative frequency, TCA relative frequency, TCC relative frequency, TCT relative frequency, TGA relative frequency, TGT relative frequency, TTA relative frequency, TTC relative frequency, TTT relative frequency |
| kNN, genetic algorithms | molecule, AG frequency, CC frequency, TA frequency, AC relative frequency, AG relative frequency, AT relative frequency, CC relative frequency, CG relative frequency, GC relative frequency, TC relative frequency, TG relative frequency, TT relative frequency, AAG relative frequency, GGA relative frequency, TGA relative frequency, TTC relative frequency |
| QDA, genetic algorithms | molecule, AA frequency, AT frequency, CT frequency, GG frequency, GT frequency, T frequency, TA frequency, TG frequency, TT frequency, AT relative frequency, CG relative frequency, TG relative frequency, AAT relative frequency, ACG relative frequency, ATC relative frequency, ATG relative frequency, ATT relative frequency, CTA relative frequency, CTC relative frequency, GGA relative frequency, TAA relative frequency, TAT relative frequency, TCT relative frequency, TGT relative frequency, TTA relative frequency, TTC relative frequency, TTG relative frequency |

*Supplementary Table ST2: Feature abundances among the feature subsets from Supplementary Table 1*

| Number of subsets in which the feature appeared | Feature name |
| --- | --- |
| 8 | molecule, GGA relative frequency |
| 7 | TG relative frequency, TAT relative frequency, TGT relative frequency, TTA relative frequency, TTC relative frequency |
| 6 | CC frequency, TA frequency, AG relative frequency, CC relative frequency, GC relative frequency, AGA relative frequency, CTA relative frequency, TTT relative frequency |
| 5 | CA frequency, CG relative frequency, AAG relative frequency, AAT relative frequency, ACG relative frequency, GAA relative frequency, GAG relative frequency, GCC relative frequency, GGC relative frequency, GGG relative frequency, GTC relative frequency, TCT relative frequency, TGA relative frequency, TGG relative frequency |
| 4 | AA frequency, AG frequency, CT frequency, GG frequency, GT frequency, TG frequency, AA relative frequency, AT relative frequency, GA relative frequency, TA relative frequency, TC relative frequency, TT relative frequency, AGC relative frequency, ATC relative frequency, ATG relative frequency, CAC relative frequency, CGT relative frequency, CTC relative frequency, GAC relative frequency, GTA relative frequency, TAC relative frequency, TAG relative frequency, TCA relative frequency, TTG relative frequency |
| 3 | AC frequency, AT frequency, CG frequency, G frequency, GA frequency, GC frequency, T frequency, AC relative frequency, CT relative frequency, GG relative frequency, AGG relative frequency, ATT relative frequency, CAT relative frequency, CCT relative frequency, CTG relative frequency, CTT relative frequency, GAT relative frequency, GCT relative frequency, GGT relative frequency, GTG relative frequency, TAA relative frequency, TCC relative frequency, TGC relative frequency |
| 2 | A frequency, TC frequency, CA relative frequency, GT relative frequency, AAA relative frequency, ACA relative frequency, ACC relative frequency, ATA relative frequency, CAA relative frequency, CAG relative frequency, CCA relative frequency, CCC relative frequency, CCG relative frequency, CGA relative frequency, CGG relative frequency, GCA relative frequency, GTT relative frequency |
| 1 | C frequency, TT frequency, AAC relative frequency, ACT relative frequency, AGT relative frequency, CGC relative frequency, GCG relative frequency, TCG relative frequency |
|  |  |


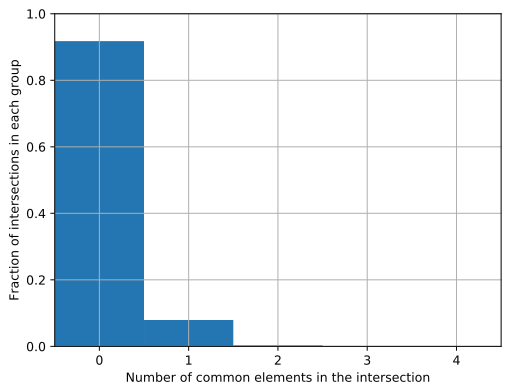


**Supplementary Figure SF1: Distribution of numbers of common elements in the feature subsets’ intersections, assuming that feature content of a subset is fully independent of feature content of other subsets.**

*Supplementary Table ST3: Feature weights after feature selection. kNN and QDA models do not evaluate feature weights.*

| *Feature name* | *Lasso* | *SelectKBEst* | *RFE* | *SCAD* | *QuiPT* |
| --- | --- | --- | --- | --- | --- |
| *molecule* | *2,561* | *0,997* | *1,292* | *1,570* | *1,130* |
| *A frequency* | *0,000* | *0,073* | *0,000* | *0,533* | *0,000* |
| *AA frequency* | *0,781* | *0,174* | *0,000* | *0,724* | *0,000* |
| *AC frequency* | *0,000* | *-0,218* | *0,000* | *0,128* | *-0,165* |
| *AG frequency* | *0,000* | *0,078* | *0,000* | *0,342* | *0,051* |
| *AT frequency* | *0,000* | *-0,025* | *0,000* | *-0,148* | *0,000* |
| *C frequency* | *0,000* | *0,000* | *0,000* | *-0,338* | *0,000* |
| *CA frequency* | *-0,933* | *-0,282* | *-0,518* | *-0,722* | *-0,345* |
| *CC frequency* | *-0,860* | *-0,313* | *-0,450* | *-1,111* | *-0,474* |
| *CG frequency* | *0,000* | *0,019* | *0,000* | *-0,112* | *0,043* |
| *CT frequency* | *0,000* | *0,088* | *0,000* | *1,572* | *-0,021* |
| *G frequency* | *0,000* | *0,035* | *0,000* | *0,285* | *0,000* |
| *GA frequency* | *0,000* | *-0,155* | *0,000* | *-0,625* | *-0,012* |
| *GC frequency* | *0,000* | *0,038* | *0,000* | *0,372* | *-0,036* |
| *GG frequency* | *0,000* | *0,109* | *0,248* | *0,781* | *0,000* |
| *GT frequency* | *0,000* | *0,014* | *0,000* | *-0,615* | *0,000* |
| *T frequency* | *0,000* | *0,067* | *0,000* | *0,000* | *0,000* |
| *TA frequency* | *0,000* | *0,145* | *0,545* | *0,000* | *0,057* |
| *TC frequency* | *0,000* | *-0,028* | *0,000* | *-0,444* | *0,000* |
| *TG frequency* | *0,000* | *-0,158* | *0,000* | *0,000* | *-0,275* |
| *TT frequency* | *0,000* | *0,000* | *0,000* | *0,000* | *0,000* |
| *AA relative frequency* | *0,000* | *-0,005* | *0,000* | *-0,389* | *0,108* |
| *AC relative frequency* | *0,000* | *0,123* | *0,000* | *0,000* | *0,000* |
| *AG relative frequency* | *0,659* | *0,286* | *0,412* | *0,000* | *0,305* |
| *AT relative frequency* | *0,000* | *0,074* | *0,000* | *0,388* | *0,000* |
| *CA relative frequency* | *0,000* | *-0,053* | *0,000* | *0,000* | *0,055* |
| *CC relative frequency* | *-1,116* | *-0,467* | *-0,512* | *-0,196* | *-0,550* |
| *CG relative frequency* | *0,000* | *0,034* | *0,000* | *0,000* | *-0,074* |
| *CT relative frequency* | *0,000* | *0,034* | *0,000* | *-0,422* | *0,005* |
| *GA relative frequency* | *-0,830* | *-0,211* | *-0,563* | *0,000* | *-0,225* |
| *GC relative frequency* | *0,865* | *0,316* | *0,511* | *0,834* | *0,312* |
| *GG relative frequency* | *0,359* | *0,061* | *0,000* | *0,000* | *0,024* |
| *GT relative frequency* | *0,000* | *0,071* | *0,000* | *0,911* | *0,000* |
| *TA relative frequency* | *-0,666* | *-0,188* | *-0,639* | *-0,947* | *0,000* |
| *TC relative frequency* | *0,000* | *0,046* | *0,000* | *0,216* | *0,000* |
| *TG relative frequency* | *-1,325* | *-0,357* | *-0,739* | *-1,073* | *-0,398* |
| *TT relative frequency* | *-0,416* | *-0,025* | *-0,296* | *0,000* | *0,000* |
| *AAA relative frequency* | *0,000* | *-0,013* | *0,000* | *0,000* | *0,081* |
| *AAC relative frequency* | *0,000* | *0,068* | *0,000* | *0,000* | *0,000* |
| *AAG relative frequency* | *0,620* | *0,301* | *0,000* | *0,374* | *0,288* |
| *AAT relative frequency* | *0,000* | *-0,039* | *0,000* | *0,109* | *-0,042* |
| *ACA relative frequency* | *0,000* | *-0,066* | *0,000* | *-0,317* | *0,000* |
| *ACC relative frequency* | *0,132* | *0,022* | *0,000* | *0,000* | *0,000* |
| *ACG relative frequency* | *0,535* | *0,150* | *0,238* | *0,431* | *0,000* |
| *ACT relative frequency* | *0,152* | *0,000* | *0,000* | *0,000* | *0,000* |
| *AGA relative frequency* | *0,597* | *0,176* | *0,247* | *0,292* | *0,398* |
| *AGC relative frequency* | *-0,213* | *-0,159* | *0,000* | *-0,447* | *0,000* |
| *AGG relative frequency* | *0,276* | *0,019* | *0,000* | *0,000* | *0,066* |
| *AGT relative frequency* | *0,000* | *0,060* | *0,000* | *0,000* | *0,000* |
| *ATA relative frequency* | *0,000* | *-0,046* | *0,000* | *-0,509* | *0,000* |
| *ATC relative frequency* | *0,298* | *0,036* | *0,000* | *0,000* | *0,000* |
| *ATG relative frequency* | *0,000* | *-0,045* | *0,000* | *0,000* | *-0,026* |
| *ATT relative frequency* | *0,000* | *-0,016* | *0,000* | *0,000* | *0,000* |
| *CAA relative frequency* | *-0,550* | *-0,006* | *0,000* | *0,000* | *0,000* |
| *CAC relative frequency* | *0,006* | *0,246* | *0,000* | *0,486* | *0,304* |
| *CAG relative frequency* | *-0,693* | *0,000* | *-0,444* | *0,000* | *0,000* |
| *CAT relative frequency* | *0,000* | *0,067* | *0,000* | *0,394* | *0,000* |
| *CCA relative frequency* | *0,000* | *0,000* | *0,000* | *-0,540* | *0,000* |
| *CCC relative frequency* | *-0,128* | *-0,100* | *0,000* | *0,000* | *0,000* |
| *CCG relative frequency* | *0,000* | *0,011* | *0,000* | *0,000* | *0,030* |
| *CCT relative frequency* | *0,496* | *0,137* | *0,000* | *0,000* | *0,128* |
| *CGA relative frequency* | *0,000* | *0,055* | *0,000* | *0,000* | *-0,079* |
| *CGC relative frequency* | *-0,080* | *0,000* | *0,000* | *0,000* | *0,000* |
| *CGG relative frequency* | *0,322* | *0,129* | *0,000* | *0,000* | *0,000* |
| *CGT relative frequency* | *0,831* | *0,334* | *0,297* | *0,556* | *0,000* |
| *CTA relative frequency* | *-0,668* | *-0,253* | *-0,300* | *-0,929* | *-0,370* |
| *CTC relative frequency* | *0,000* | *-0,056* | *0,000* | *0,000* | *-0,095* |
| *CTG relative frequency* | *-0,200* | *-0,117* | *0,000* | *0,000* | *-0,060* |
| *CTT relative frequency* | *0,000* | *-0,062* | *0,000* | *0,000* | *-0,212* |
| *GAA relative frequency* | *0,437* | *0,091* | *0,412* | *-0,039* | *0,069* |
| *GAC relative frequency* | *-0,473* | *-0,125* | *0,000* | *-0,605* | *-0,271* |
| *GAG relative frequency* | *0,000* | *-0,084* | *-0,131* | *-0,532* | *-0,080* |
| *GAT relative frequency* | *0,294* | *0,166* | *0,400* | *0,000* | *0,000* |
| *GCA relative frequency* | *0,000* | *-0,019* | *0,000* | *-0,117* | *0,000* |
| *GCC relative frequency* | *-0,846* | *-0,263* | *-0,272* | *-0,541* | *-0,230* |
| *GCG relative frequency* | *0,000* | *-0,060* | *0,000* | *0,000* | *0,000* |
| *GCT relative frequency* | *-0,225* | *-0,007* | *0,000* | *0,000* | *-0,073* |
| *GGA relative frequency* | *-0,511* | *-0,270* | *-0,475* | *-0,404* | *-0,390* |
| *GGC relative frequency* | *-0,950* | *-0,338* | *-0,466* | *-0,954* | *-0,416* |
| *GGG relative frequency* | *0,000* | *-0,006* | *-0,234* | *-0,475* | *-0,076* |
| *GGT relative frequency* | *0,000* | *0,147* | *0,000* | *0,000* | *-0,165* |
| *GTA relative frequency* | *0,325* | *0,163* | *0,000* | *-0,226* | *0,171* |
| *GTC relative frequency* | *-0,466* | *-0,159* | *-0,291* | *-0,619* | *-0,218* |
| *GTG relative frequency* | *0,000* | *-0,140* | *0,000* | *-0,411* | *0,000* |
| *GTT relative frequency* | *0,000* | *0,112* | *0,000* | *0,000* | *0,000* |
| *TAA relative frequency* | *0,000* | *0,106* | *0,000* | *0,000* | *0,000* |
| *TAC relative frequency* | *0,225* | *0,133* | *0,000* | *0,246* | *0,190* |
| *TAG relative frequency* | *-0,603* | *-0,188* | *-0,591* | *0,000* | *-0,176* |
| *TAT relative frequency* | *0,648* | *0,212* | *0,337* | *0,439* | *0,221* |
| *TCA relative frequency* | *-0,236* | *0,107* | *0,000* | *-0,407* | *0,000* |
| *TCC relative frequency* | *0,000* | *0,084* | *0,000* | *0,000* | *0,202* |
| *TCG relative frequency* | *0,000* | *0,038* | *0,000* | *0,000* | *0,000* |
| *TCT relative frequency* | *0,000* | *0,099* | *0,000* | *-0,342* | *0,084* |
| *TGA relative frequency* | *0,000* | *0,145* | *0,000* | *-0,325* | *0,034* |
| *TGC relative frequency* | *0,000* | *-0,068* | *0,000* | *-0,431* | *0,059* |
| *TGG relative frequency* | *-0,996* | *-0,281* | *-0,604* | *-0,888* | *-0,385* |
| *TGT relative frequency* | *-0,733* | *-0,150* | *-0,361* | *-0,879* | *-0,375* |
| *TTA relative frequency* | *-0,845* | *-0,283* | *-0,819* | *-1,066* | *-0,409* |
| *TTC relative frequency* | *1,148* | *0,333* | *0,000* | *0,371* | *0,476* |
| *TTG relative frequency* | *-0,515* | *-0,256* | *-0,552* | *0,000* | *0,000* |
| *TTT relative frequency* | *-0,422* | *-0,123* | *-0,443* | *-0,412* | *-0,092* |

*
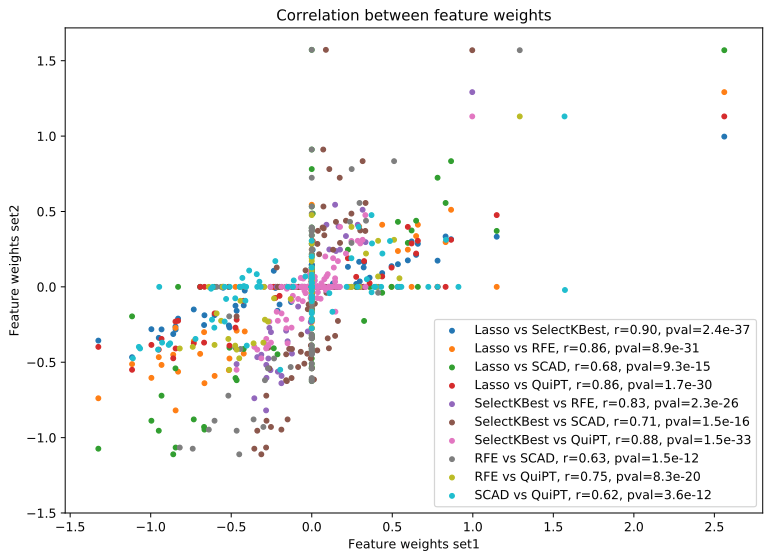
*

**Supplementary Figure SF2: Correlations between feature weights for feature sets obtained with various feature selection approaches. The perpendicular lines centered at (0, 0) are an effect of select feature importances after feature selection being set to 0. For each pair of feature sets, Pearson correlation coefficient and 2-tailed p-value are given.**

*Supplementary Table ST4: Number of object from each class when evaluating HTP's performance on subsequences obtained from new viral sequences*

| Length | Substitution rate | Number of subsequences | |
| --- | --- | --- | --- |
|  |  | Eukaryotic viruses | Phages |
| 100 | 0,00 | 58855 | 41145 |
|  | 0,02 | 58840 | 41160 |
| 250 | 0,00 | 58428 | 41572 |
|  | 0,02 | 58539 | 41461 |
| 500 | 0,00 | 58028 | 41972 |
|  | 0,02 | 58204 | 41796 |
| 1000 | 0,00 | 57158 | 42842 |
|  | 0,02 | 57429 | 42571 |
| 3000 | 0,00 | 54423 | 45577 |
|  | 0,02 | 54446 | 45554 |
| 10000 | 0,00 | 51612 | 48388 |
|  | 0,02 | 51519 | 48481 |

*Supplementary Table ST5: AUC calculated on subsequences derived from new eukaryotic viruses*

| Length of a subsequence | AUC | | | | | | | |
| --- | --- | --- | --- | --- | --- | --- | --- | --- |
|  | Substitution rate = 0 | | | | Substitution rate = 0.02 | | | |
|  | LR | SVC | kNN | QDA | LR | SVC | kNN | QDA |
| 100 | 0,604 | 0,541 | 0,595 | 0,500 | 0,604 | 0,538 | 0,596 | 0,500 |
| 250 | 0,668 | 0,596 | 0,661 | 0,525 | 0,671 | 0,601 | 0,665 | 0,525 |
| 500 | 0,721 | 0,671 | 0,719 | 0,536 | 0,724 | 0,676 | 0,722 | 0,536 |
| 1000 | 0,763 | 0,745 | 0,768 | 0,569 | 0,764 | 0,745 | 0,767 | 0,567 |
| 3000 | 0,791 | 0,837 | 0,804 | 0,709 | 0,791 | 0,835 | 0,800 | 0,707 |
| 10000 | 0,793 | 0,910 | 0,822 | 0,904 | 0,793 | 0,910 | 0,823 | 0,904 |


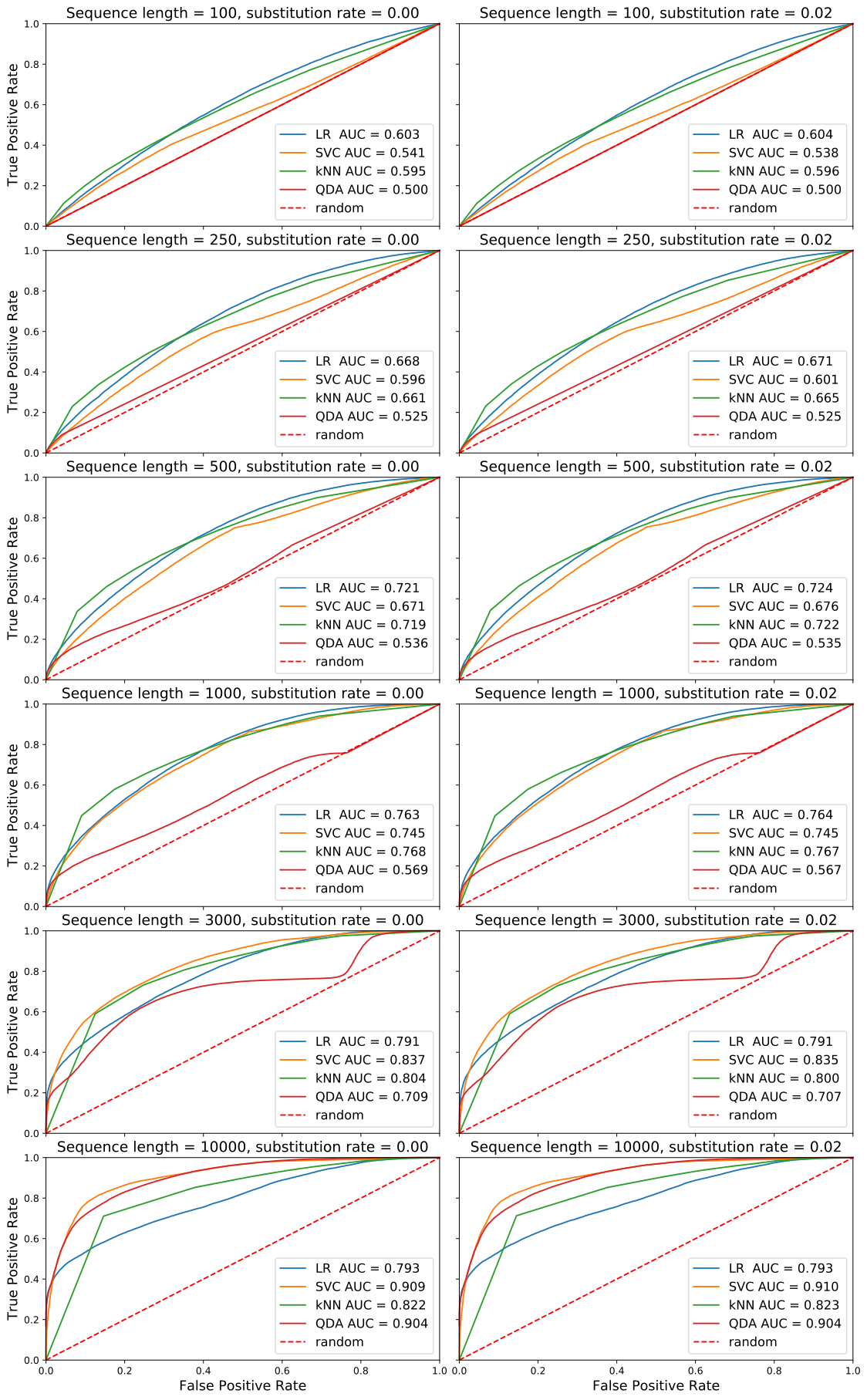


**Supplementary Figure SF3. Receiver operating characteristics curves for HTP’s performance evaluation on subsequences obtained from newly published viral genomic sequences. TPR and FPR values essential to compute the AUCs are available in json format at** [**https://github.com/wojciech-galan/supplementary_materials/blob/master/datasets/results_for_stimulated_metagenomics.json**](https://github.com/wojciech-galan/supplementary_materials/blob/master/datasets/results_for_stimulated_metagenomics.json)

**
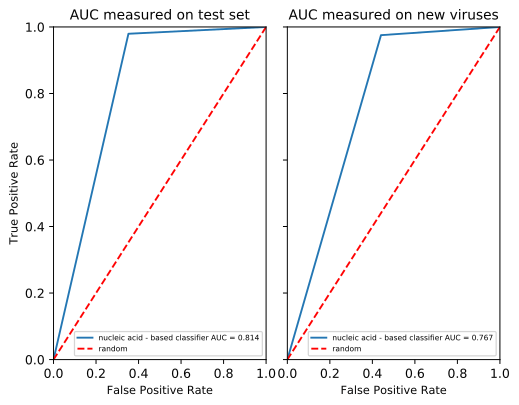
**

**Supplementary Figure SF4. Receiver operating characteristics curves for classification solely based on nucleic acid type (DNA viruses classified as phages, RNA viruses as eukaryotic).**

**
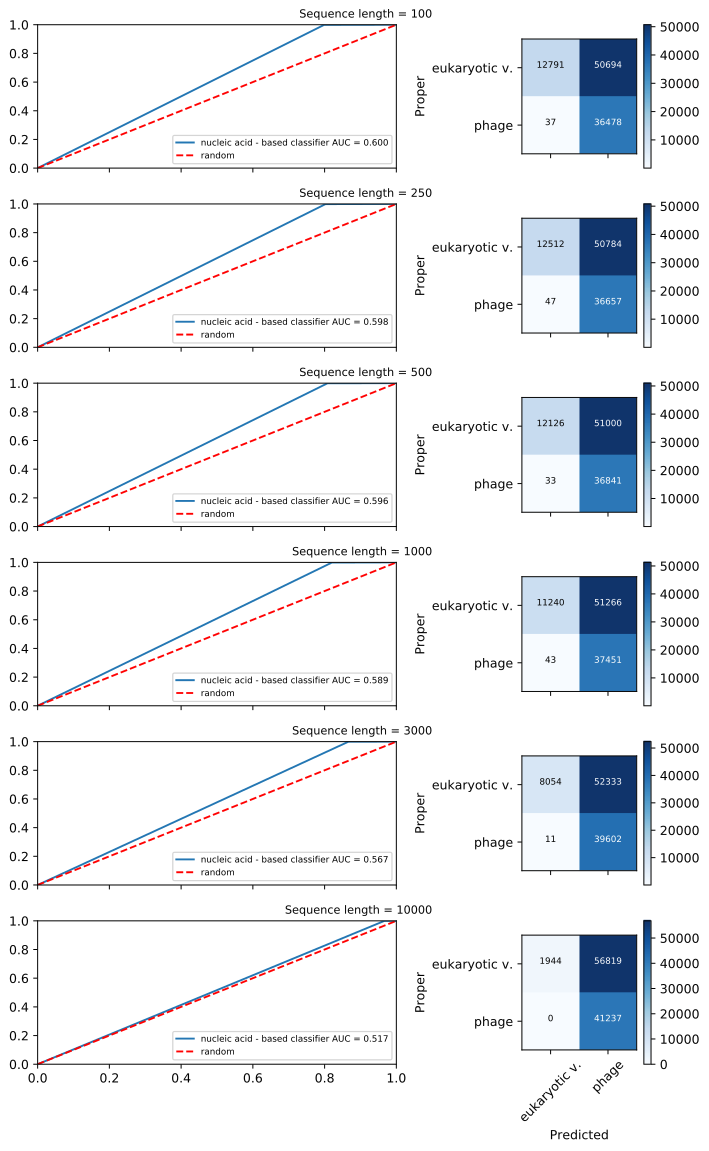
**

**Supplementary Figure SF5. Receiver operating characteristics curves when sequences obtained from newly published viral genomic subsequences are classified solely based on nucleic acid type (left) along with corresponding confusion matrices (right). Subsequences originating from DNA particles were classified as obtained from phages  while the ones originating from RNA - as obtained from eukaryotic viruses. With increasing subsequence length, percentage of DNA subsequences originating from eukaryotic viruses (thus incorrectly classified as originating from phages) in our dataset also increases.**

**Supplementary Notes:**

**Data partitioning (eukaryotic viruses vs phages):**

Phages of lineages containing phrases *'Microviridae', 'Cystovirus', 'Allolevivirus', 'Picovirinae', 'P22virus', 'Autographivirinae', 'Phietavirus', 'Sk1virus', 'Andromedavirus', 'unclassified archaeal viruses', 'unclassified bacterial viruses', 'Peduovirinae', 'Vequintavirinae'* were assigned to the test set. Eukaryotic viruses of lineages containing phrases *'Virus families not assigned to an order', 'Virus-associated RNAs', 'unclassified ssRNA viruses', 'Togaviridae', 'Idaeovirus', 'Nodaviridae', 'Dicistroviridae', 'Ophioviridae', 'Pneumoviridae', 'Filoviridae', 'Bornaviridae', 'Arenaviridae', 'Gammatorquevirus', 'Babuvirus', 'unclassified Geminiviridae', 'Densovirinae', 'unclassified Parvoviridae', 'Curtovirus', 'Turncurtovirus', 'Hypoviridae', 'Aquareovirus', 'Nudiviridae', 'Lambdapapillomavirus', 'Aviadenovirus', 'Iridoviridae', 'Betaherpesvirinae', 'unclassified dsDNA viruses', 'unclassified Betapolyomavirus', 'Babu- and nanovirus-associated alphasatellites', 'Double-stranded RNA satellites', 'Hepadnaviridae', 'Gemykibivirus'* were handled similarly. The remaining viruses were assigned to the cross-validation set. (Subsequently, the removal of select eukaryotic viruses was carried out in order for the set to contain an identical number of phages and eukaryotic viruses). Identifiers of the viral sequences assigned to cross-validation and test set are available at <https://github.com/wojciech-galan/supplementary_materials/blob/master/datasets/ids_learn> and <https://github.com/wojciech-galan/supplementary_materials/blob/master/datasets/ids_test>, respectively. Indices of viruses assigned to each cross-validation split are available at <https://github.com/wojciech-galan/supplementary_materials/blob/master/datasets/cv_indices>.

**Data partitioning (subgroups of eukaryotic viruses):**

Viruses of lineages containing phrases: *'Single stranded DNA satellites', 'Nanoviridae', 'Anelloviridae', 'unclassified ssDNA viruses', 'Virgaviridae', 'Bromoviridae', 'Sobemovirus', 'Picornavirales', 'Nodaviridae', 'Mononegavirales', 'Ophioviridae', 'unclassified Reoviridae', 'Spinareovirinae', 'Picobirnaviridae', 'Totiviridae', 'Adenoviridae', 'Baculoviridae'* were assigned to the test set. Identifiers of the viral sequences assigned to cross-validation and test set are available at <https://github.com/wojciech-galan/supplementary_materials/blob/master/datasets/extension_ids_learn> and <https://github.com/wojciech-galan/supplementary_materials/blob/master/datasets/extension_ids_test>, respectively. Indices of viruses assigned to each cross-validation split are available at <https://github.com/wojciech-galan/supplementary_materials/blob/master/datasets/extension_cv_indices>.

**Default classifiers’ parameters (sklearn):**

kNN: *n_neighbors=5*, *weights=’uniform’*, *algorithm=’auto’*, *leaf_size=30*, *p=2*, *metric=’minkowski’*, *metric_params=None*

QDA: *priors=None*, *reg_param=0.0*, *store_covariance=False*, *tol=0.0001*, *store_covariances=None*

SVC: *C=1.0*, *kernel=’linear’*, *degree=3*, *gamma=’auto_deprecated’*, *coef0=0.0*, *shrinking=True*, *probability=False*, *tol=0.001*, *cache_size=200*, *class_weight=None*, *verbose=False*, *max_iter=-1*, *decision_function_shape=’ovr’*

LR: *C=1.0*, *kernel=’rbf’*, *degree=3*, *gamma=’auto_deprecated’*, *coef0=0.0*, *shrinking=True*, *probability=False*, *tol=0.001*, *cache_size=200*, *class_weight=None*, *verbose=False*, *max_iter=-1*, *decision_function_shape=’ovr’*

**Genetic Algorithms setup:**

Three types of crossover were used in the study. Initially, we used one-point, two-point, and uniform crossover. In final run, the latter was solely used based on results from our initial and extended analyses indicating uniform crossover method as yielding high-scoring feature sets most efficiently.

Initial settings for kNN and QDA (100 individuals in the population, up to 200 rounds of selection, elitist selection - the fittest individual was copied directly to the next generation):

| mutation probabilities* | tournament size | features** | neighbors*** | three types of crossover |
| --- | --- | --- | --- | --- |
| 0.05, 0.075, 0.1, 0.125, 0.15, 0.175. 0.2 | 2, 4, 6, 8 | 10, 20, 30, 40, 50, 60, 70, 80, 90 | 1, 3, 5, 7 | YES |

extended analysis - kNN (100 individuals in the population, up to 200 rounds of selection):

| mutation probabilities* | tournament size | features** | neighbors | crossover type |
| --- | --- | --- | --- | --- |
| 0.05, 0.075, 0.1, 0.125, 0.15, 0.175. 0.2 | 2, 4, 6, 8 | 10, 20 | 9, 11, 13, 15 | uniform |
| 0.25, 0.3 | 2, 4, 6, 8 | 10, 20 | 1, 3, 5, 7, 9, 11, 13, 15 | uniform |

extended analysis - QDA (100 individuals in the population, up to 200 rounds of selection):

| mutation* probabilities | tournament size | features** | three types of crossover |
| --- | --- | --- | --- |
| 0.25, 0.3 | 2, 4, 6, 8 | 10, 20, 30, 40, 50, 60, 70, 80, 90 | YES |

final settings (500 individuals in the population, up to 200 rounds, elitist selection, and uniform crossover only):

kNN:

| mutation probabilities* | tournament size | features** | neighbors |
| --- | --- | --- | --- |
| 0.05, 0.075, 0.1, 0.125, 0.15, 0.175, 0.2 | 2 | 10, 20 | 7, 11, 13, 15 |
| 0.05, 0.075, 0.1, 0.125, 0.15, 0.175, 0.2 | 4 | 10, 20 | 7, 9, 11, 13, 15 |
| 0.05, 0.075, 0.1, 0.125, 0.15, 0.175, 0.2 | 6 | 10, 20 | 7, 9, 11 |
| 0.05, 0.075, 0.1, 0.125, 0.15, 0.175, 0.2 | 8 | 10, 20 | 9, 11 |

QDA:

| mutation probabilities* | tournament size | features** |
| --- | --- | --- |
| 0.15, 0.175, 0.2, 0.25, 0.3 | 2, 4, 6 | 20, 30, 40, 50, 60 |

* Mutation defined as flipping one bit in the binary vector while mutation probability of m is the probability of flipping each of the individual’s attributes equaling $\frac{m}{number of attributes}$

** features of n corresponds to the exact number of features in an individual taken from a normal distribution with mean=n and sd=5.

*** applies to kNN only

**Inclusion of longer oligonucleotides to our feature set:**

The absolute tetranucleotide frequencies were included in the feature set, which resulted in 101 + 256 = 357 features. We compared SVC classifier’s with default parameters (RBF kernel, C = 1,$=\frac{1}{number of features}$) performance trained on feature sets with and without tetranucleotides. The larger feature set demonstrate better prediction capabilities (‘fitness’ as measured on cross-validation data equals 1.94 compared to 1.90 for the basic feature set, similarly AUC as measured on the test set equals 0.990 versus 0.988). The comparison was performed with our script available at <https://github.com/wojciech-galan/supplementary_materials/blob/master/code/normal_vs_tetra.py>


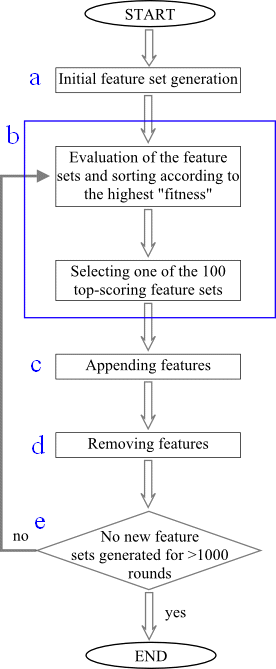


**Supplementary Figure SF6: Bottom-up feature selection algorithm:**

**a). Feature sets were evaluated using 10-fold stratified cross-validation, and final ‘fitness’ value for each feature set was computed according to the equation 3 (see Methods section).**

**b). One of 100 top-scoring feature sets was picked in a manner that favors the higher scoring ones: the probability that the top-scoring feature set will be selected equals
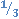
. The total probability of randomly selecting one of next 9 feature sets equals
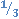
, thus each of those feature sets could be picked with probability of
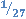
. A similar rule applies to remaining 90 feature sets: the total probability of selecting one of sets equals
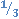
, so each of the feature sets could be selected with probability of
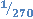
 . The selected feature set was then used as a basis for appending features.**

**c). Not-already-contained features are appended to the set. Assuming that there are 102 possible features, and that a set already contains 3 of them, 99 new four-element sets are created.**

**d). In the first iteration, the algorithm moves directly to a step ‘e’ because feature set contains no more than four features, and all of shorter feature sets have already been evaluated. Starting from the second iteration of the algorithm, some not-yet-evaluated feature sets may appear. Assuming that in the first iteration, the set (A, B, C) was picked for appending features, sets (A, B, C, *, where * is any of the remaining features) were generated in step ‘c’ and then, in the second iteration, set(A, B, C, D) selected for appending features. One of sets generated in this manner was (A, B, C, D, E). Its select subsets haven’t yet been evaluated: (B, C, D, E), (A, C, D, E), (A, B, D, E), (A, B, C, E), and will be evaluated in this step.**

**e). Feature sets generated by the algorithm in steps ‘a’, ‘c’, and ‘d’ are stored in a collection. When a feature set is selected in step ‘b’ for the second time, no new feature sets are generated in steps ‘c’ and ‘d’, and collection remains unchanged. When the algorithm is close to converging, most of the top-scoring feature sets had already been selected for appending features, so the collection changes very rarely.**
